# Is Care Partner Burden Associated With Negative Post-Acute Outcomes in Older Adults With Dementia?

**DOI:** 10.1093/geroni/igaf122.1959

**Published:** 2025-12-31

**Authors:** Diane Berish

**Affiliations:** Pennsylvania State University, University Park, Pennsylvania, United States

## Abstract

Informal care partners are asked to care for individuals living with dementia with little formal training or supports. During the period after hospitalization, care partners face multiple demands, including increased care dependency of the person with dementia and increased likelihood for adverse events in the care receiver. This secondary analysis of Fam-FFC trial data examined the association between care partner burden, both level and change, and negative outcomes, including falls, emergency department transfers, injuries, and hospital admissions, following acute care hospitalization in a sample of 339 care partner/care recipient dyads. The average age for care partners was 61 years and 81 years for care recipients. Most care partners were female (72%) and married (61%). Burden, assessed using the Zarit Short Form, increased from discharge (M = 9.2, SD = 9.0) to two-months (M = 12.6, SD = 9.9) with average change being 3.5 (SD = 8.7, p < 0.0001). Emergency room visits were the most frequent negative outcome at 27% (93/339), followed by falls (24%, 83/339), hospitalizations (21%, 72/339) and injuries (12%, 40/339). Logistic regression models were created for level at discharge and change in care partner burden (discharge to two-months) predicting negative outcomes 6-months post discharge. Intervention effects, care partner, and care recipient factors were included as control variables. No measures of care partner burden were predictive of negative outcomes at 6-months. These findings suggest that the first two months after discharge may be a critical period when caregiver burden intensifies, highlighting the importance of early support to help caregivers navigate this transition and maintain their ability to provide care.

